# Ten Year Results of Extensive Nodal Radiotherapy and Moderately Hypofractionated Simultaneous Integrated Boost in Unfavorable Intermediate-, High-, and Very High-Risk Prostate Cancer

**DOI:** 10.3390/cancers13194970

**Published:** 2021-10-03

**Authors:** Nadia Gisella Di Muzio, Chiara Lucrezia Deantoni, Chiara Brombin, Claudio Fiorino, Cesare Cozzarini, Flavia Zerbetto, Paola Mangili, Roberta Tummineri, Italo Dell’Oca, Sara Broggi, Marcella Pasetti, Anna Chiara, Paola Maria Vittoria Rancoita, Antonella Del Vecchio, Mariaclelia Stefania Di Serio, Andrei Fodor

**Affiliations:** 1 Department of Radiotherapy, IRCCS San Raffaele Scientific Institute, 60 Olgettina Street, 20132 Milan, Italy; deantoni.chiaralucrezia@hsr.it (C.L.D.); cozzarini.cesare@hsr.it (C.C.); zerbetto.flavia@hsr.it (F.Z.); tummineri.roberta@hsr.it (R.T.); delloca.italo@hsr.it (I.D.); pasetti.marcella@hsr.it (M.P.); chiara.anna@hsr.it (A.C.); fodor.andrei@hsr.it (A.F.); 2 Vita-Salute San Raffaele University, 20132 Milan, Italy; brombin.chiara@hsr.it (C.B.); rancoita.paolamaria@unisr.it (P.M.V.R.); clelia.diserio@unisr.it (M.S.D.S.); 3 University Center for Statistics in the Biomedical Sciences, Vita-Salute San Raffaele University, 58 Olgettina Street, 20132 Milan, Italy; 4 Medical Physics, IRCCS San Raffaele Scientific Institute, 20132 Milan, Italy; fiorino.claudio@hsr.it (C.F.); mangili.paola@hsr.it (P.M.); broggi.sara@hsr.it (S.B.); delvecchio.antonella@hsr.it (A.D.V.)

**Keywords:** radiotherapy in high-risk prostate cancer, moderately hypofractionated radiotherapy, prostate cancer radiotherapy boost, pelvic radiotherapy in prostate cancer, ADT and radiotherapy in prostate cancer

## Abstract

**Simple Summary:**

Several phase III randomized trials of moderate hypofractionation, including a higher proportion of high-risk prostate cancer patients treated only to the prostate, failed to demonstrate the superiority of hypofractionated regimens. There is only one randomized phase III trial, of moderately hypofractionated high-dose radiotherapy to the prostate-only versus pelvic irradiation and prostate boost, with a sufficiently long follow-up. It demonstrated better biochemical and disease-free survival when lymph nodal radiotherapy was added. Here we present the 10-year results of our experience based on an Institutional protocol adopted after a phase I–II study, on patients with unfavorable intermediate- (UIR), high- (HR), and very high-risk (VHR) prostate cancer (PCa) treated with pelvic lymph nodal irradiation (WPRT) and moderately hypofractionated high-dose (HD) simultaneous integrated boost (SIB) to the prostate. Prognostic factors for relapse, as well as acute and late gastro-intestinal (GI) and genito-urinary (GU) toxicity were also analyzed.

**Abstract:**

Aims: To report 10-year outcomes of WPRT and HD moderately hypofractionated SIB to the prostate in UIR, HR, and VHR PCa. Methods: From 11/2005 to 12/2015, 224 UIR, HR, and VHR PCa patients underwent WPRT at 51.8 Gy/28 fractions and SIB at 74.2 Gy (EQD2 88 Gy) to the prostate. Androgen deprivation therapy (ADT) was prescribed in up to 86.2% of patients. Results: Median follow-up was 96.3 months (IQR: 71–124.7). Median age was 75 years (IQR: 71.3–78.1). At last follow up, G3 GI–GU toxicity was 3.1% and 8%, respectively. Ten-year biochemical relapse-free survival (bRFS) was 79.8% (95% CI: 72.3–88.1%), disease-free survival (DFS) 87.8% (95% CI: 81.7–94.3%), overall survival (OS) 65.7% (95% CI: 58.2–74.1%), and prostate cancer-specific survival (PCSS) 94.9% (95% CI: 91.0–99.0%). Only two patients presented local relapse. At univariate analysis, VHR vs. UIR was found to be a significant risk factor for biochemical relapse (HR: 2.8, 95% CI: 1.17–6.67, *p* = 0.021). After model selection, only Gleason Score ≥ 8 emerged as a significant factor for biochemical relapse (HR = 2.3, 95% CI: 1.12–4.9, *p* = 0.023). Previous TURP (HR = 3.5, 95% CI: 1.62–7.54, *p* = 0.001) and acute toxicity ≥ G2 (HR = 3.1, 95% CI = 1.45–6.52, *p* = 0.003) were significant risk factors for GU toxicity ≥ G3. Hypertension was a significant factor for GI toxicity ≥ G3 (HR = 3.63, 95% CI: 1.06–12.46, *p* = 0.041). ADT (HR = 0.31, 95% CI: 0.12–0.8, *p* = 0.015) and iPsa (HR = 0.37, 95% CI: 0.16–0.83, *p* = 0.0164) played a protective role. Conclusions: WPRT and HD SIB to the prostate combined with long-term ADT, in HR PCa, determine good outcomes with acceptable toxicity.

## 1. Introduction

External beam radiotherapy for prostate cancer has changed dramatically over the past two decades. As time has progressed, dose escalation studies have brought conventional fractionation regimens to 74–80 Gy using 1.8 to 2.0 Gy fractions, which has been shown to achieve greater biochemical disease control when compared to 64–70.2 Gy [1,2,3,4,5,6]. Given the radiobiological understanding of prostate cancer radiation dose response to larger fraction sizes, as well as the prolonged treatment course required to deliver modern doses of external beam radiotherapy, individual institutions and cooperative groups have developed an interest in using larger fraction sizes for treatment. Large clinical trials have tested moderate hypofractionation, including CHHiP, RTOG 0415, PROFIT, and HYPRO. Some of these studies were designed as non-inferiority trials [7,8,9,10,11] and have now been reported with a minimum of 5 years of follow-up data [8,9,10,11,12,13,14,15,16]. Although all these studies included localized or locally advanced prostate cancer, differences were present in the patient population in terms of the T stage, Gleason score, pretreatment prostate-specific antigen level, risk category, use of androgen deprivation therapy (ADT), irradiated portals, and radiation therapy (RT) schedules (i.e., total dose, dose/fraction, number of fractions, and overall treatment time) in both regimens. Outcomes are usually reported as biochemical failure (BF), biochemical and/or clinical failure (BCF), overall survival (OS), prostate cancer-specific survival (PCSS), and early and late gastrointestinal (GI) and genitourinary (GU) toxicity. With the publication of these four large phase III studies on moderate hypofractionation, it is now considered safe and effective [8,10,11,12,17,18], and guidelines from the American Society for Radiation Oncology (ASTRO), American Society of Clinical Oncology (ASCO), and American Urological Association (AUA) state that moderate hypofractionation should be offered to low-risk, intermediate-risk, and high-risk localized prostate cancer candidates for external beam radiotherapy (EBRT) [7]. However, with the exception of a subset of patients in the Fox Chase trial [13], none of the studies referred to by the guidelines included radiation of an elective pelvic nodal field, and long-term efficacy data beyond 5 years are still lacking. The role of whole pelvic RT (WPRT) in high-risk localized prostate cancer may be associated with a risk of occult pelvic lymph node metastases as high as 40% [19]. Such patients are currently treated with a combination of high dose radiation and long course ADT. The benefit of prophylactic regional nodal irradiation in high-risk cases is yet to be determined conclusively, even after two randomized trials [20,21]. However radiotherapy dose and delivery techniques in these trials may be considered less than optimal by current standards.

Our department has a long tradition of dose-escalation and WPRT [22,23]. The purpose of this analysis is to present long-term outcomes and toxicities of our protocol, adopted after a prospective phase I–II study of WPRT using ADT and image guided IMRT with a hypofractionated simultaneous integrated boost (SIB) to the prostate in unfavorable intermediate-, high-, and very high-risk prostate cancer.

## 2. Materials and Methods

In November 2004, after the installation of the first TomoTherapy^®^ (Accuray, Sunnyvale, CA, USA) system in our department, we started numerous studies on moderate hypofractionation. On 3 November 2005, a phase I–II, open label prospective clinical trial of moderate hypofractionated prostate cancer irradiation with IG-IMRT was approved by the institutional ethics committee (DS/URC/ER/mm prot. no. 714/DG). This protocol has been described in previously published papers [24,25] and due to the good results combined with other reports on moderate hypofractionation was adopted as standard treatment in our department. All treated patients signed an informed consent both for treatment and publication of disease related information, in accordance with the Helsinki declaration. All patients included in the intermediate- and high-risk groups at that time were reclassified according to NCCN v2019 as favorable intermediate-risk (FIR: Grade Group 2 and no other risk factors, not included in this analysis), unfavorable intermediate-risk (UIR: with two or three intermediate risk factors between T2b–T2c, Grade Group 2 and PSA 10–20 ng/mL, and/or Grade Group 3 and/or ≥50% biopsy cores positive), high-risk (HR: T3a, or Grade Group 4 or 5, or PSA > 20 ng/mL), or very high-risk-group (VHR: T3b–T4, or primary Gleason pattern 5, or >4 cores with Grade Group 4 or 5) [26]. The outcomes of 224 UIR, HR, and VHR patients treated with this protocol up to December 2015 are analyzed in this study. TNM-staging was mainly done by digital rectal examination, transrectal US, bone scan, and a diagnostic thoracic and abdomino-pelvic computed tomographic (CT) scan. Patients with distant metastatic disease were excluded. Briefly, four target volumes (PTVs) were defined receiving different dose levels, with the highest dose level administered as SIB. In parentheses, the 2 Gy equivalent dose (EQD2) for α/β 1.5 is shown:

1. PTV prostate: 74.2 Gy in 28 fractions (fr), (EQD2: 88 Gy);
2. PTV prostate and caudal seminal vesicles (SVs): 65.5 Gy in 28 fr, (EQD2: 72 Gy) in UIR and 74.2 Gy in 28 fr for HR and VHR patients. The overlap between rectum and prostate PTV was constrained to 65.5 Gy;
3. PTV prostate and caudal + proximal SVs: 61.6 Gy in 28 fr, (EQD2: 65 Gy);
4. PTV prostate, SVs, and pelvic lymph nodes (common iliac (under L5-S1 space/aortic bifurcation after 2012), external iliac, internal iliac, presacral, and obturator lymph nodes): 51.8 Gy in 28 fr, (EQD2: 50 Gy).

ADT primarily consisted of an oral anti-androgen or gonadotropin-releasing hormone agonist. All patients received luteinizing hormone-releasing hormone analogs 3–5 months before initiation of radiation therapy and with the addition of an antiandrogen the first 2–4 weeks to prevent a tumor flare.

The primary endpoints were peak Radiation Therapy Oncology Group (RTOG)/National Cancer Institute Common Terminology Criteria for Adverse Events toxicity scale, version 5.0 (CTCAE) [27] acute and late GU tract and GI tract toxicity. Secondary endpoints were biochemical recurrence free survival (bRFS), OS, disease free survival (DFS), and PCSS. Additionally, we evaluated the best cut-off value for initial PSA (iPSA) allowing the best distinction among different OS outcomes.

Details of planning and image guidance have been reported in other publications [25,28,29]. In short, patients’ legs were immobilized in the supine position with a Combifix^TM^ (CIVCO Radiotherapy). A pelvic planning CT was acquired with a 3–5 mm slice thickness, and an empty rectum and full bladder were required. All patients received WPRT (with the lymph nodal clinical target volume (CTV) extended up to at least the L5-S1 intervertebral space in the first years, and then to aortic bifurcation). An SIB to the prostate and the proximal third of SVs was delivered. The lymph nodal planning target volume (PTV) encompassed the lymph nodal CTV with a margin of 7 mm, while prostate and SVs PTV had an 8 mm margin in all directions except for cranio-caudal, which was 10 mm. Original planning parameters and all other dosimetric constraints have been detailed previously [28,30]. A daily mega-voltage computed tomography (MVCT) was performed for all patients, with active rectal evacuation or bladder filling, in order to ensure a precise IG radiotherapy [29].

### 2.1. Patient Population

Patient characteristics are shown in Table 1. One hundred and ninety-three (86.2%) patients underwent ADT as follows: 51(70.8%) UIR, 54 (87.1%) HR, 88 (97.8%) VHR. Neo-adjuvant ADT was prescribed in 186 patients (83%) for a median of 3.55 months (2.45–5.31), and adjuvant ADT in 181 patients (80.8%) for a median of 22.71 months (13.69–31.70). According to the NCCN risk group classification, the median treatment period was 22.25 months (11.97–28.23) in UIR, 27.95 months (17.66–38.59) in HR, and 31.70 months (23.68–40.80) in VHR. Some comorbidities were evaluated in relation to patient toxicity and outcomes: diabetes, hypertension, abdominal surgery, and previous TURP.

### 2.2. Follow-Up: Outcome and Toxicity Evaluation

Toxicity (physician-reported) was prospectively evaluated during treatment and at each follow-up visit. Patients were visited every week, starting from the second week, during the treatment, and appointments were scheduled every three to six months in the first year and every six months for the next two years, with PSA performed every three months, and annually up to the tenth year thereafter, with PSA performed every six months. GU and GI toxicities were evaluated using the National Cancer Institute Common Terminology Criteria for Adverse Events toxicity scale (CTC-AE) version 5.0. The time to development of the worst-grade toxicity was documented as was the symptom resolution, either spontaneous or subsequent to medical treatment/surgical procedure. Biochemical relapse was calculated using the Phoenix Consensus Conference definition (nadir + 2 ng/mL) [31]. Local and distant failures were defined on image-based (PET/MR/TC) or histologically-confirmed evidence of prostate cancer. Prostate cancer mortality was defined as death due to prostate cancer progression or with elevated PSA during salvage therapies.

### 2.3. Statistical Analysis

Median and IQR were used as summary statistics to describe continuous variables, while categorical variables were expressed as frequency and percentage. The Kaplan–Meier method was used to estimate bRFS and DFS from the end of radiotherapy, OS, from the diagnosis, and PCSS, from the diagnosis. A log-rank test was used to compare survival of groups of patients. A survival tree (ST) analysis was performed to identify the best cut-off value for initial PSA, allowing the best distinction among different OS outcomes. After testing whether the proportional hazards assumption was met, using both inferential procedures and graphical diagnostics, univariate and multivariate Cox regression analyses were performed on the survival outcomes of interest considering as covariates the following variables: age at diagnosis, T stage, Gleason score, diabetes, hypertension, previous abdominal surgery, hormonal therapy, and the categorized version of the initial PSA (based on the cut-off value selected by ST analysis). Backward selection procedures were applied to identify a smaller set of relevant covariates significantly associated with the outcomes. The same statistical analyses were applied to identify risk/protective factors for grade ≥3 late GU or GI toxicity, considering the same covariates as in the models described above; the only difference was that of entering iPsa as a continuous log-transformed variable instead of using it in its categorized version. In addition, the role of previous TURP, grade ≥2 acute toxicity GU and a variable indicating the combined maximum toxicity between acute rectal toxicity and acute upper GE were evaluated. Estimated hazard ratios (HR) along with 95% confidence intervals were reported. All the analyses were performed using R statistical software (version 3.5.2, https://cran.r-project.org/index.html) (accessed on 15 June 2021). In all the analyses, the significance level was set at 0.05.

## 3. Results

### 3.1. Outcomes

**Figure 1:**
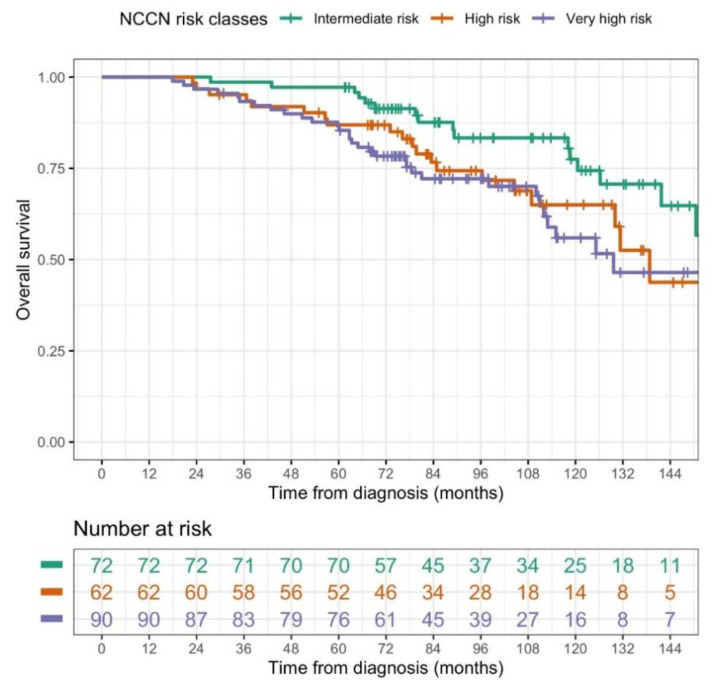
Kaplan–Meier estimates of overall survival (OS, computed from the diagnosis) in the three NCCN risk classes (*p* = 0.096, log-rank test; NCCN risk class VHR vs. NCCN risk class UIR, HR = 1.8792, 95% CI: 1.0509–3.3604, *p* = 0.03338, univariate Cox regression model). Although all the data were used for statistical analyses, here, for graphic purposes only, the plot was curtailed at 12 years, since the proportion of patients experiencing the event after this time was negligible.

**Figure 2:**
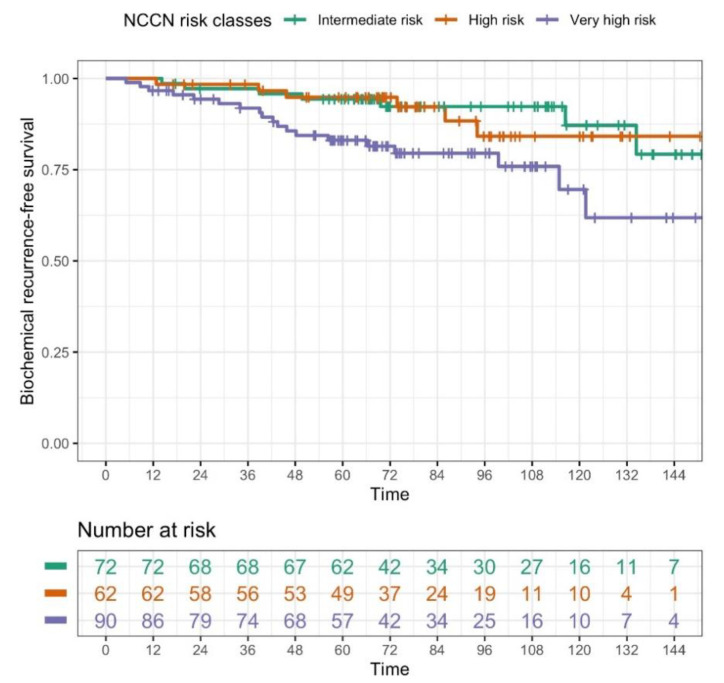
Kaplan–Meier estimates for biochemical relapse-free survival (bRFS) in the three NCCN risk classes (*p* = 0.021, log-rank test; NCCN risk class VHR vs. NCCN risk class UIR, HR = 2.7924, 95% CI: 1.1689–6.6705, *p* = 0.0208, univariate Cox regression model). Although all the data were used for statistical analyses, here, for graphic purposes only, the plot was curtailed at 12 years, since the proportion of patients experiencing the event after this time was negligible.

All 224 patients completed treatment as planned and were alive at least 90 days after the completion of radiation treatment for the evaluation of late toxicity and outcomes. Median follow-up was 96.3 months (IQR: 71.0–124.6) from the end of RT and 107.6 months (IQR: 78.35;136.10) from the diagnosis. Median age at diagnosis was 74.9 years (IQR: 71.3–78.1). Thirty-two out of 224 patients presented a biochemical relapse during the follow-up. Only two patients presented a local relapse, and 18 a distant relapse. Seventy-one patients were dead at the last follow-up (May 2021), 8 from prostate cancer, 9 from other tumors (1 lung, 2 colon, 1 gastric, 1 myeloid leukemia, 1 liver, 1 larynx, and 2 brain), 45 for other causes, and 9 not specified (lost to follow-up with date of death known, but not the cause). Patients dead from prostate cancer were one UIR, one HR, and six VHR. Five- and 10-year median OS from diagnosis were 90.1% (95% CI: (86.3–94.1%)) and 65.7% (95% CI: (58.2–74.1%)), respectively. Five- and 10-year bRFS were 90.1% (95% CI: (86.1–94.2%)) and 79.8% (95% CI: (72.3–88.1%)), while DFS was 92.3% (95% CI: (88.7–96.0%)) at 5 years and 87.8% (95% CI: (81.7–94.3%)) at 10 years. PCSS at 5 at 10 years was 99% ((95% CI: (97.7–100%)) and 94.9% (95% CI: (91.0–99.0%)), respectively (the nine patients with not specified cause of death were excluded from this latter analysis). There was no statistically significant difference in the OS (considering time from diagnosis) between the three risk groups (see Figure 1), but VHR patients had a significantly (*p* = 0.021) worse biochemical control (see Figure 2). Log-rank test highlighted a significant difference in the biochemical control of the three groups. Moreover, in the post hoc analysis involving pairwise comparisons between groups using the log-rank test, the biochemical control for VHR patients significantly differed from that of unfavorable intermediate-risk patients, (*p* = 0.046, after Bonferroni’s correction). Five- and 10-year outcomes are reported in Table 2.

A cut-off of 18 ng/mL of initial PSA was found as the first split in survival trees for OS outcomes (considering either time from diagnosis or time from the end of RT). At Cox univariate analysis, only age at diagnosis (HR 1.095, 95% CI: 1.0425–1.1503, *p* = 0.0003) and NCCN risk classification (NCCN risk class VHR vs. NCCN risk class UIR, HR = 1.8792, 95% CI: 1.0509–3.3604, *p* = 0.03338) emerged as significant risk factors for OS (considering time from diagnosis). For biochemical relapse, GS score (GS score ≥ 8 vs. GS score ≤ 7, HR: 2.3349, 95% CI: 1.1221–4.8587, *p* = 0.0233), and NCCN risk classification (NCCN risk class VHR vs. NCCN risk class UIR, HR = 2.7924, 95% CI: 1.1689–6.6705, *p* = 0.0208) were found to be significant risk factors. Similar findings were reported when examining DFS (univariate Cox regression model): GS score (GS score ≥ 8 vs. GS score ≤ 7, HR = 3.6137, 95% CI: 1.3091–9.9755, *p* = 0.0132) and NCCN risk classification (NCCN risk class VHR vs. NCCN risk class UIR, HR = 3.4757, 95% CI: 1.1395–10.602, *p* = 0.0286) emerged as significant risk factors. With reference to the multivariate model, after model selection, age at diagnosis (HR = 1.10941, 95% CI: 1.055–1.167, *p* < 0.001), iPSA ≥ 18 ng/mL (HR = 1.86174, 95% CI 1.104–3.141, *p* = 0.0198) and T stage (T3/T4 vs. T1/T2, HR = 2.07127, 95% CI: 1.131–3.793, *p* = 0.0183) emerged as significant risk factors for OS (considering time from diagnosis). Again, considering the final selected model, only Gleason score emerged as significant risk factor for biochemical relapse (GS score ≥ 8 vs. GS score ≤ 7, HR = 2.3349, 95% CI: 1.122–4.859, *p* = 0.0233) and DFS (GS score ≥ 8 vs. GS score ≤ 7, HR = 3.6137; 95% CI: 1.309–9.975; *p* = 0.0131). For complete results, see Table 3.

### 3.2. Toxicity

The crude incidence of acute and late toxicity as well as the prevalence of late toxicity at the last follow-up are reported in Table 4 according to RTOG/CTCAE v5 scales. Late GI and GU toxicity improved spontaneously, with drugs or interventions (argon plasma coagulation for actinic proctitis or transurethral prostatic incision (TUIP) for urethral stenosis); thus, at the last follow-up G3 GI toxicity had decreased from 8.5% to 3.1%, and GU ≥ G3 toxicity from 12.5% to 8%. Three patients presented G4 events; one patient presented urethrostomy after repeated catheterization for acute urinary retention, and the other two were cystectomized, both with hematuria and tight stenosis, requiring repeated TUIP, with subsequent complete incontinence.

**Figure 3:**
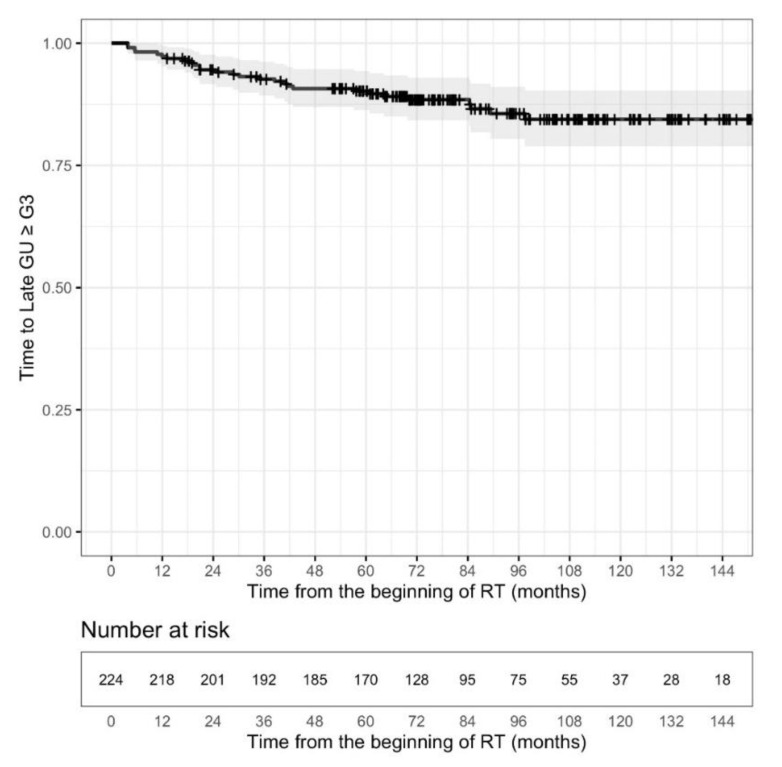
Kaplan–Meier estimate of time to late GU ≥ G3 toxicity. Although all the data were used for statistical analyses, here, for graphic purposes only, the plot was curtailed at 12 years.

**Figure 4:**
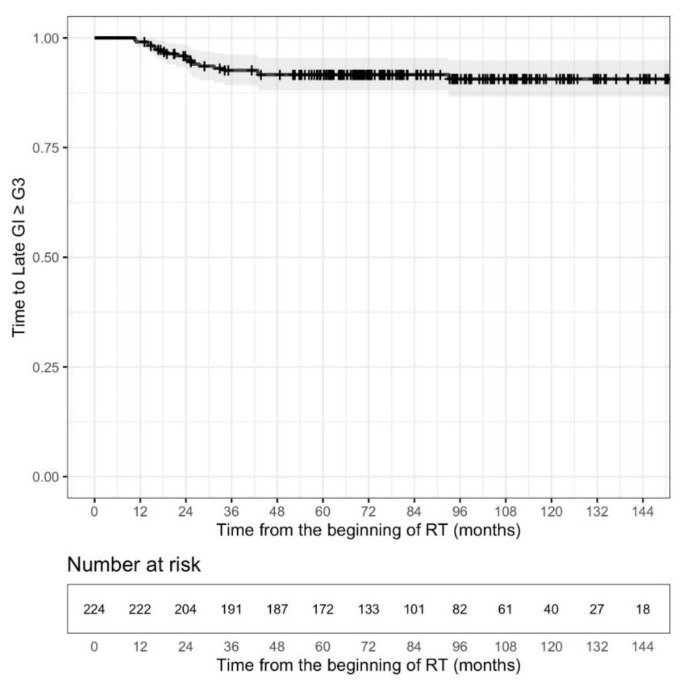
Kaplan–Meier estimate of time to late GI ≥ G3. Although all the data were used for statistical analyses, here, for graphic purposes only, the plot was curtailed at 12 years.

Freedom from significant GU (≥G3) toxicity at 10 years was estimated to be 84.4% (95% CI: 78.9–90.3). A plateau was registered approximately 108 months after the end of treatment (see Figure 3). Freedom from late GI ≥ G3 toxicity at 10 years was estimated to be 90.6% (95% CI: 86.6–94.9); a plateau was reached at approximately 48 months, earlier than that observed for late GU (see Figure 4). At the univariate level, acute GU toxicity ≥ G2 (HR = 2.6187, 95% CI: 1.248–5.494, *p* = 0.0109), and previous TURP (HR = 2.9464, 95% CI: 1.38–6.293, *p* = 0.00526) were found to be significant risk factors for late GU ≥ G3, while adjuvant ADT (HR = 0.4326, 95% CI: 0.1952–0.9585, *p* = 0.039) was associated with a significant reduction of the risk of late GU ≥ G3. Initial PSA, on log scale (HR = 0.3642, 95% CI: 0.1739–0.7626, *p* = 0.00739), neo-adjuvant ADT (HR = 0.2189, 95% CI: 0.08891–0.5392, *p* = 0.00096), ADT (HR = 0.2632, 95% CI: 0.1036–0.669, *p* = 0.00503), adjuvant ADT (HR = 0.2411, 95% CI: 0.0978–0.5944, *p* = 0.002) were associated, at the univariate level, with a significant reduction of the risk of late GI ≥ G3. Hypertension was retained in the final selected model playing the role of risk factor for late GI ≥ G3 toxicity (HR = 3.6287, 95% CI: 1.0567–12.4610, *p* = 0.0406), while iPSA (on a logarithmic scale) and hormonal therapy were found to be protective factors (HR = 0.3677, 95% CI: 0.1624–0.8326, *p* = 0.0164 and HR = 0.3104, 95% CI: 0.1208–0.7974, *p* = 0.0151). For complete analysis, see Table 5.

## 4. Discussion

When considered in total, the body of work investigating the safety and efficacy of moderate hypofractionation vs. conventional fractionation in the treatment of prostate cancer strongly supports its equivalence in terms of outcomes with the added benefits of decreased costs and increased patient convenience. Nine randomized trials reported from 2005 to 2017, including a total of 8146 patients, were considered as a reference for evaluating the efficacy of moderately hypofractionated treatment [8,9,10,11,12,13,14,15,16], given their minimum follow-up period of 5 years. Although the relationship between the freedom from BF (FFBF) and PCSS is not yet understood, the primary outcome measure reported by all published randomized trials of hypofractionated RT (HFRT) versus conventional fractionated RT (CFRT) has been FFBF. BF, defined as the elevation of the prostate-specific antigen (PSA) beyond a threshold of 2 ng/mL after the nadir following a radiation treatment, is a marker of disease relapse in both loco-regional and/or distant sites. Nonetheless, owing to both the exhaustion of the protective action of concomitant/adjuvant ADT, and the clinical development of incidental pre-treatment micro-metastases within 5 years, 5-year FFBF can be taken as an optimal surrogate endpoint for local tumor control and an acceptable measure of radiation effectiveness [32].

The outcome data of the abovementioned trials, published in a meta-analysis by a Swiss group showed that 20.6% and 18.0% of the HFRT patients experienced bRFS and clinical relapse-free survival (CRFS), respectively [33]. Prostate cancer specific mortality (PCSM) was documented in 1.9% of the HFRT groups [33]. Our data, although our findings refer to a longer follow-up, 10 years, and to only HR prostate cancer patients, compare favorably with these data, considering that 5- and 10-year bRFS were 90.1% and 79.8%, while DFS (CRFS) was 92.3% at 5 years and 87.8% at 10 years. PCSS at 5 and 10 years was 99% and 94.9%, respectively. The Arcangeli et al. study [9], included in the aforementioned meta-analysis, reported 72% of 10-year FFBF in 83 HR patients treated with 62 Gy/20 fractions delivered in 5 weeks. All patients received 9 months of ADT; median follow-up was 9 years. The HYPRO trial that enrolled patients with IR to HR prostate cancer also reported relapse-free survival at 7 years in 71.7% HR patients [34].

A small single institution study recently reported 10-year results after image guided, intensity modulated radiation therapy with hypofractionated simultaneous integrated boost and elective pelvic fields [35]. In the 82 HR-VHR treated patients, 10-year bRFS was 64%, PCSS 90%, and OS 72%. There were 11 patients with local recurrence in the total cohort; local recurrence occurred in 14% of the VHR group. Our study registered only two local relapses. The 10-year outcomes of one of the first studies of moderate hypofractionation for patients treated with intensity modulated radiation therapy (IMRT) for localized prostate cancer at 70 Gy in 28 fractions, at 2.5 Gy per fraction was recently reported. Considering only UIR and HR patients, the outcome data are significantly less favorable compared to our data, especially for bRFS 71% in UIR and 42% in HR patients: CRFS 85% in UIR and 72% in HR and PCSM 5% in UIR and 15% in HR [36]. The authors reported their decision to continue to offer hypofractionated IMRT for HR patients, while increasing ADT prescription to a minimum of 2 years, with the addition (in many cases) of RT to the pelvic lymph nodes at 50.4 Gy in 28 fractions (at 1.8 Gy per fraction), while simultaneously treating the prostate and proximal seminal vesicles at 70 Gy [36].

Two published randomized trials from the nineties previously explored the benefit of adding pelvic radiotherapy for localized prostate cancer [20,21]. A post hoc subgroup analysis of the GETUG-01 trial after 11 years of follow-up favored pelvic radiotherapy in patients with <15% Roach nodal risk [21]. Although initial results of the RTOG 9413 trial suggested improved biochemical control with WPRT, long term outcomes have shown no clear difference between PORT (prostate only RT) and WPRT.

POP-RT is a randomized single institution trial comparing PORT and WPRT in patients with HR, node negative prostate cancer. Dose prescription was 68 Gy in 25 fractions to the prostate in the PORT arm, and 50 Gy in 25 fractions to the pelvic nodes in the WPRT arm, with an SIB to 68 Gy to the prostate. Recently they reported the preliminary outcome results: 5-year bRFS was 95.0% in the WPRT arm and 81.2% in the PORT arm, respectively, with an unadjusted HR for BF of 0.23 (95% CI, 0.10 to 0.52, *p* < 0.0001) favoring WPRT. Competing risk analysis for the primary end point also showed a significant difference in the cumulative incidence of primary events, favoring WPRT (5% vs. 19%, Gray’s test, *p* < 0.0001) [37]. The long-term results of the POP-RT trial clearly show that the failure events in the PORT arm starts at about 36 months, corresponding to the recovery of testosterone in these patients and suggesting the necessity of WPRT for the long-term control of microscopic disease in regional nodes. This study was similar to ours in terms of RT pelvic volume and RT dose to pelvic lymph nodes, and, after a median follow up of 10 years, in our study we registered the metastatic spread only in 18 patients (8%).

Again our results compare favorably with the 5-year OS of 92% and bRFS of 87% reported for HR prostate cancer patients treated with ADT and 20-fraction HFRT delivered to the prostate and pelvic nodal areas by a Canadian group [38].

The majority of our patients received neo adjuvant and concomitant ADT to RT, prolonging the assumption for a median of 27 months. In the largest reported analysis of WPRT for patients with HR prostate cancer treated in the dose-escalated era, Amini et al. indicated that the addition of WPRT demonstrated no survival advantage compared with POP-RT [39]. On the other hand, Lawton et al. reported an unexpected interaction between the timing of hormonal therapy and radiation field size for HR patient population [40]. His paper certainly could represent an additional argument in favor of WPRT + NHT (neo-adjuvant hormonal therapy) that would correspond with the results of RTOG 92-02 and 86-10 trials [41,42]. This analysis showed a clear benefit in both biochemical control and PFS in favor of WPRT. In fact, when comparing the WPRT + NHT arm vs. PORT + NHT arm, a trend is seen toward statistical significance in PFS (*p* = 0.066), and biochemical failure using the Phoenix definition (*p* = 0.0098). This suggests that if one chooses to use NHT for this population of prostate cancer patients, WPRT appears to provide a benefit compared with PORT. One possible explanation for the benefit of WPRT + NHT compared with WPRT + AHT could lie in the immune modulation of antiandrogen ablation therapy, resulting in T-cell infiltration of the prostate before and during RT, increasing apoptosis, and making RT more effective at the doses used to treat the lymph nodes [43]. More recently, a meta-regression of 40 individual trials with 21,429 total patients suggested that the advantage of long-term ADT exceeds that of increasing the radiation dose alone [44]. However, the same authors reported that the small number of trials utilizing high dose RT and short course ADT limits the capability to detect differences between this treatment and others. In fact, no statistical differences in 5-year outcomes were found when a threshold of 76 Gy was used for high dose RT.

Three ongoing trials have addressed the issue of radiation therapy on pelvic nodes but are likely to have definitive results only in the next 10 years: RTOG 0924, Pivotal Boost (patients with UIR and favorable HR), the French GETUG AFU-23 trial (on unfavorable HR patients, but is a 2 × 2 factorial design studying the value of neoadjuvant cabazitaxel in addition to WPRT).

In our study, only Gleason score ≥8 emerged as a significant risk factor for biochemical relapse and distant relapse, while a cut-off of 18 ng/mL of initial PSA was found for OS outcomes. For Gleason score 9–10 prostate cancer, in a retrospective cohort study involving 12 centers, with 1809 patients treated between 2000 and 2013 with radical prostatectomy (RP), EBRT with ADT, or EBRT plus brachytherapy boost (BT) and ADT, Kishan et al. observed that the best PCSM and time to distant metastasis were obtained in patients treated with EBRT + BT + ADT, despite the significantly shorter duration of ADT. Patients treated with EBRT and doses to prostate ≥ 78 Gy + 24 months of ADT also had better outcomes than patients treated with RP [45]. Martinez, using a dose escalation protocol with a BT boost to the prostate, reported a 10-year BF rate of 18.9%, clinical failure rate of 7.7%, and distant metastasis of 5.7% in patients treated with BED > 268 Gy (α/β = 1.2) [46]. We administered an SIB with photons delivering a BED of 238 Gy (α/β = 1.2), obtaining overlapping results. More recently Wedde et al. reported that HR prostate cancer has a significantly reduced PCSM and overall mortality (OM) rates when treated with dose-escalated radiotherapy achieved by EBRT + HDR-BT compared to EBRT alone (70 Gy), confirming the need for high dose to obtain better tumor control [47].

In the meta-analysis of nine randomized trials on moderate hypofractionation [33], acute and late GU morbidities consistently showed no significant differences, and no heterogeneity was observed among the studies. The lack of significant differences for either acute or late GU morbidity may have been registered due to the fact that treatment portals in nearly all studies were confined to the prostate with or without SVs. Late GI and GU toxicity incidences were not significantly different. On the contrary the incidence of acute GI toxicity and the heterogeneity in both acute and late GI effects significantly increased. Our results are consistent with other prospective trials of moderate hypofractionated radiotherapy in terms of GI and GU toxicity ≥ grade 2. Considering the study with the longest follow-up (11.3 years) [36], the reported data of late toxicity showed a 10-year cumulative incidence rate of Grade >3 late GU toxicity of 2% and GI late Grade > 3 toxicity of 1%. In our study, freedom from significant GU (≥G3) toxicity at 10 years was estimated to be 84.4%. A plateau was registered at approximately 9 years after the end of treatment. Freedom from late GI ≥ G3 toxicity at 10 years was estimated to be 90.6%; a plateau was reached at approximately 4 years, earlier than that observed for late GU. At the last follow-up, G3 GI toxicity decreased from 8.5% to 3.1%, and GU ≥ G3 toxicity from 12.5% to 8%. WPRT delivered with hypofractionated IG-IMRT resulted in increased G2 or higher late GU toxicity as compared to PORT in the POP-RT trial. With a median follow-up of 68 months, cumulative > G2 late GU toxicity was significantly higher with WPRT (20.0% vs. 8.9%, *p* = 0.02), while no statistically significant difference was observed for > G2 late GI toxicity (8.2% vs. 4.5%, *p* = 0.28). Dosimetric analysis showed higher bladder volume receiving 30–40 Gy in the WPRT arm [37]. These data are consistent with our results. Saracino et al. [48] published the 5-year results in 110 HR patients treated with pelvic IMRT and SIB to the prostate area. The 3- and 5-year grade ≥ 2 late rectal toxicities were 2% and 5%, respectively, whereas the 3- and 5-year late GU toxicity grades ≥2 were 5% and 12%, respectively. Unfortunately there is little data regarding clinical predictors of toxicity that might allow improved patient selection for hypofractionated treatment. We confirm after 10 years of follow up that in our study, the acute GU toxicity > grade 2 and TURP seem to be the only predictors of late GU toxicity. Lawton et al., in the update of the RTOG 94-13 trial, reported no difference in acute radiation toxicity ≥ G3, worse acute hormonal toxicity with neoadjuvant ADT, similar late GU toxicity, and a statistically significant increase in GI ≥ G3 toxicity in the neoadjuvant ADT+ WPRT arm vs. the other arms, including WPRT+ adjuvant ADT [40]. Unlike the result of the randomized DART01/05 GICOR trial [49], reporting that long-term ADT did not significantly impact urinary or rectal radiation-induced toxicity; in our experience, adjuvant ADT is associated with a significant reduction of the risk of late GU ≥ G3, while lymph node irradiation was not identified as a risk factor for GI toxicity. Hypertension was retained, playing the role of risk factor for late GI ≥ G3 toxicity, and iPSA (on a logarithmic scale) was found to be a protective factor.

Concerns regarding higher bowel toxicity with WPRT were addressed with the use of helical IMRT to optimize small bowel sparing. It also allowed the safe inclusion of common iliac nodes within the pelvic treatment volume as compared to many trials that limited their pelvic field portals to the L5/S1 or S1/S2 vertebral junction, possibly missing a substantial proportion of the lymph nodes draining the prostate [50,51].

The main strengths of the study are the prospective study design, the long follow-up, and the high number of patients homogeneously treated with modern techniques in a single institution. A follow-up period beyond 5 years is critical when analyzing long-term endpoints, such as late GU toxicity, PCSM, bRFS, and local recurrence. Although patients were enrolled between 2005 and 2015, treatment techniques with image guided IMRT, dose-escalation, hypofractionation, and SIB are up to date according to the guidelines. The absence of a central pathology evaluation is a study limitation. Reports on tolerance were based on physician reports rather than from patient reported data. Hence, there was a risk of underreported toxicity, but the results were re-evaluated by two physicians in order to better interpret them from the perspective of the new CTCAE vs. 5.0 toxicity scale. Despite the use of IG-IMRT, CTV–PTV margins remained the same as in our previous 3D-CRT protocols, which certainly increased the overall toxicity, thus not taking full advantage of the possibilities offered by technology and worsening the overall toxicity results.

The analyzed patients treated with this protocol were all enrolled between 2005 and 2015, when PET/CT was not considered a suitable examination for initial staging. Thus, all were staged with thoracic and abdominal contrast-enhanced computed tomography and bone scintigraphy. Given the high specificity of PSMA PET/CT observed in prospective studies, a positive PSMA PET in a few lymph nodes could change the therapeutic strategy with the addition of a simultaneous integrated boost to improve lymph nodal control, or, in the case of extensive metastases, to refer the patient to exclusive systemic therapies, thus improving the patient selection. Given the low sensitivity of only 40%, a negative PSMA PET/CT at the initial staging cannot represent the justification for reducing dissection or treatment volumes in high and very high-risk disease [52,53].

SBRT is currently consolidating its position as a valid treatment option for the prostate, having the advantage of a much shorter duration, with up to seven fractions delivered in a maximum of two weeks. The HYPO-RT-PC trial demonstrated the non-inferiority of this approach versus conventionally fractionated radiotherapy, even though only 11% of patients were at high-risk [54]. Based on several meta-analyses of prospective phase I–II studies, including some high-risk patients, NCCN (National Comprehensive Cancer Network) guidelines recently approved this treatment for high-risk cancers as well [55,56]. The addition of pelvic lymph nodal irradiation remains an open issue. Therefore, the final decision will remain in the hands of the prescribing physician.

In addition to the clinical and histopathological variables analyzed in our series, genetic variables could have a crucial role. Krebs et al. demonstrated a VEGFR2 upregulation in the high-risk clinical setting, and Norby et al. claimed a correlation between VEGFR2 expression and biochemical and clinical progression. Genomic biomarkers such as Decipher, Oncotype DX, and Prolaris could be useful tools to stratify low-risk from high-risk tumors and guide personalized treatment decisions [57,58].

## 5. Conclusions

Our study showed that WPRT, with HD moderately hypofractionated SIB to prostate and SVs, and long term ADT in UIR, HR, and VHR PCa patients obtained good bRFS and DFS, with acceptable toxicity. Previous TURP and acute toxicity ≥ G2 predicted GU toxicity ≥ G3, while hypertension predicted GI toxicity ≥ G3. ADT was a protective factor for GI toxicity. Only GS score was determinant for bRFS and DFS. This update after 10 years of median follow-up confirms the more than acceptable results in terms of both toxicity and clinical outcomes. The improved bRFS may reflect the synergy of a very high EQD2 dose, treatment of pelvic lymph nodes, and careful daily image guidance procedures.

A possible randomized prospective study between prostate only SBRT vs. hypofractionated pelvic and prostate irradiation both with long and short ADT, including biomarkers, modern imaging such as PSMA PET/CT (which could help in patient selection, excluding those already metastatic), using reduced margins (therefore reducing volumes and subsequently the toxicity), based on the precision offered by the daily IGRT, could help to obtain a more precise answer to the Hamletic “pelvis yes/pelvis no” doubt.

## Figures and Tables

**Table 1:** Patients and treatment characteristics.

		Stratified by NCCN Risk Class		
	Overall	4	5	6
n	224	72	62	90
Age diagnosis (median (IQR))	74.99 (71.31, 78.13)	75.73 (71.95, 78.04)	73.40 (69.25, 76.54)	76.23 (72.42, 78.99)
T (%)				
T1a	2 (0.9)	1 (1.4)	1 (1.6)	0 (0.0)
T1c	70 (31.2)	27 (37.5)	22 (35.5)	21 (23.3)
T2/3	1 (0.4)	0 (0.0)	1 (1.6)	0 (0.0)
T2a	24 (10.7)	11 (15.3)	6 (9.7)	7 (7.8)
T2b	8 (3.6)	2 (2.8)	2 (3.2)	4 (4.4)
T2c	82 (36.6)	31 (43.1)	18 (29.0)	33 (36.7)
T3	17 (7.6)	0 (0.0)	5 (8.1)	12 (13.3)
T3a	9 (4.0)	0 (0.0)	7 (11.3)	2 (2.2)
T3b	10 (4.5)	0 (0.0)	0 (0.0)	10 (11.1)
T4	1 (0.4)	0 (0.0)	0 (0.0)	1 (1.1)
iPsa (median (IQR))	10.80 (6.54, 17.73)	9.36 (6.31, 12.88)	20.00 (6.89, 26.59)	10.89 (6.48, 16.66)
Gleason score (%)				
6	20 (8.9)	6 (8.3)	14 (22.6)	0 (0.0)
3 + 4	31 (13.8)	16 (22.2)	15 (24.2)	0 (0.0)
4 + 3	60 (26.8)	50 (69.4)	10 (16.1)	0 (0.0)
sum 8	68 (30.4)	0 (0.0)	17 (27.4)	51 (56.7)
sum 9	40 (17.9)	0 (0.0)	5 (8.1)	35 (38.9)
sum 10	4 (1.8)	0 (0.0)	0 (0.0)	4 (4.4)
N/A	1 (0.4)	0 (0.0)	1 (1.6)	0 (0.0)
NCCN risk class (%)				
UIR	72 (32.1)			
HR	62 (27.7)			
VHR	90 (40.2)			
Neoadjuvant ADT	186 (83.0)	50 (69.4)	50 (80.6)	86 (95.6)
Androgen Deprivation Therapy (ADT)	193 (86.2)	51 (70.8)	54 (87.1)	88 (97.8)
Adjuvant ADT	181 (80.8)	43 (59.7)	51 (82.3)	87 (96.7)
Duration Neoadjuvant ADT (median (IQR))	3.55 (2.45, 5.31)	3.37 (2.32, 5.00)	3.92 (2.85, 7.50)	3.44 (2.45, 4.91)
Duration Adjuvant ADT (median (IQR))	22.71 (13.69, 31.70)	18.91 (9.07, 24.10)	22.91 (14.00, 30.03)	26.21 (17.49, 33.47)
Duration ADT (median (IQR))	27.17 (18.74, 37.82)	22.25 (11.97, 28.23)	27.95 (17.66, 38.59)	31.70 (23.68, 40.80)
Diabetes (%)	30 (13.4)	4 (5.6)	15 (24.2)	11 (12.2)
Hypertension (%)	137 (61.2)	43 (59.7)	38 (61.3)	56 (62.2)
Abdominal Surgery (%)	101 (45.1)	27 (37.5)	28 (45.2)	46 (51.1)
Previous TURP (%)	42 (18.8)	11 (15.3)	14 (22.6)	17 (18.9)

IQR = interquartile range, T = tumor, iPSA = initial prostatic specific antigen, ADT = androgen deprivation therapy, TURP = transurethral prostate resection.

**Table 2:** Five- and 10-year biochemical relapse—(bRFS), disease free—(DFS), overall—(OS), and -prostate cancer-specific survival (PCSS) in percentages with 95% confidence intervals (CIs). Kaplan–Meier estimates were reported for all patients and within NCCN risk classes. PCSS stratified analysis was not performed due to the small number of events.

Kaplan Meier Estimates	All Patients % (95% CI)	Unfavorable Intermediate-Risk % (95% CI)	High-Risk % (95% CI)	Very High-Risk % (95% CI)
5-year bRFS	90.1% (86.1–94.2)	94.3% (89.1–99.9)	94.8% (89.3–100)	83.1% (75.3–91.6)
10-year bRFS	79.8% (72.3–88.1)	87.2% (76.3–99.6)	84.2% (72.4–97.9)	69.6% (55.5–87.1)
5-year DFS	92.3% (88.7–96.0)	95.8% (91.2–100)	96.3% (91.4–100)	86.4% (79.2–94.2)
10-year DFS	87.8% (81.7–94.3)	90.7% (80.7–100)	96.3% (91.4–100)	79.8% (69.2–92.1)
5-year OS	90.1% (86.3–94.1)	97.2% (93.5–100)	86.9% (78.8–95.8)	86.5% (79.7–93.9)
10-year OS	65.7% (58.2–74.1)	77.5% (66.4–90.4)	65.0% (52.1–81.2)	55.9% (43.7–71.7)
5-year PCSS	99 (97.7–100)			
10-year PCSS	94.9% (91.0–99.0)			

**Table 3:** Univariate and multivariate analysis of factors influencing outcomes.

**OS from Diagnosis**	**Univariate (Cox Regression Model)**		Multivariate (Final * Selected Cox Regression Model)	
Variables	HR (95% CI)	*p*-value	HR (95% CI)	*p*-value
Age at diagnosis	1.095 (1.0425–1.1503)	**0.0003**	1.1094 (1.0551–1.1665)	**0.0001**
T stage T3/T4 (ref: T1 + T2)	1.6001 (0.8879–2.8837)	0.1178	2.0713 (1.1312–3.7926)	**0.0183**
Gleason ≥ 8 (ref: ≤7)	1.5449 (0.9619–2.4813)	0.072	-	-
ADT (yes vs. no)	2.2967 (0.8347–6.3195)	0.1074	-	-
Abdominal surgery (yes vs. no)	1.2161 (0.7575–1.9523)	0.4179	-	-
Hypertension (yes vs. no)	0.7402 (0.4592–1.1933)	0.217	-	-
Diabetes (yes vs. no)	0.9215 (0.4404–1.9281)	0.8281	-	-
iPsa ≥ 18 (ref: <18)	1.4624 (0.8758–2.4419)	0.1463	1.8617 (1.1036–3.1407)	**0.0198**
Neoadjuvant ADT (yes vs. no)	2.2252 (0.8926–5.5473)	0.0861		
Adjuvant ADT (yes vs. no)	1.2689 (0.6266–2.5694)	0.5083		
NCCN risk class 5 (ref: class 4)	1.5735 (0.8307–2.9805)	0.1642		
NCCN risk class 6 (ref: class 4)	1.8792 (1.0509–3.3604)	**0.0334**		
**bRFS from the End of RT**	**Univariate (Cox Regression Model)**		Multivariate (Final * Selected Cox Regression model)	
Variables	HR (95% CI)	*p*-value	HR (95% CI)	*p*-value
Age at diagnosis	1.0627 (0.9913–1.1392)	0.0867	-	-
T stage T3/T4 (ref: T1 + T2)	1.3399 (0.5507–3.2601)	0.519	-	-
Gleason ≥ 8 (ref: ≤7)	2.3349 (1.1221–4.8587)	**0.0233**	2.3349 (1.1221–4.8587)	**0.0233**
ADT (yes vs. no)	1.0474 (0.3662–2.9958)	0.9312	-	-
Abdominal surgery (yes vs. no)	0.9091 (0.4506–1.834)	0.7901	-	-
Hypertension (yes vs. no)	1.8542 (0.8327–4.1289)	0.1306	-	-
Diabetes (yes vs. no)	1.7129 (0.7023–4.1779)	0.2368	-	-
iPsa ≥ 18 (ref: <18)	0.998 (0.4306–2.3129)	0.9962	-	-
Neoadjuvant ADT (yes vs. no)	0.81 (0.332–1.9761)	0.6432		
Adjuvant ADT (yes vs. no)	1.0986 (0.421–2.8663)	0.8477		
NCCN risk class 5 (ref: class 4)	1.1474 (0.3843–3.4259)	0.8054		
NCCN risk class 6 (ref: class 4)	2.7924 (1.1689–6.6705)	**0.0208**		
**DFS from the End of RT**	**Univariate (Cox Regression Model)**		Multivariate (Final * Selected Cox Regression model)	
Variables	HR (95% CI)	*p*-value	HR (95% CI)	*p*-value
Age at diagnosis	1.0918 (0.9955–1.1974)	0.0623	-	-
T stage T3/T4 (ref: T1 + T2)	1.9339 (0.7021–5.3265)	0.202	-	-
Gleason ≥ 8 (ref: ≤7)	3.6137 (1.3091–9.9755)	**0.0132**	3.6137 (1.3091–9.9755)	**0.0131**
ADT (yes vs. no)	3.1606 (0.4227–23.6309)	0.2623	-	-
Abdominal surgery (yes vs. no)	0.7333 (0.2978–1.806)	0.5	-	-
Hypertension (yes vs. no)	1.7864 (0.6489-4.918)	0.2615	-	-
Diabetes (yes vs. no)	1.2207 (0.357-4.1743)	0.7506	-	-
iPsa ≥ 18 (ref: <18)	0.8679 (0.2899-2.5987)	0.8001	-	-
Neoadjuvant ADT (yes vs. no)	1.8775 (0.4351-8.1017)	0.3985		
Adjuvant ADT (yes vs. no)	2.0281 (0.4697-8.7579)	0.3434		
NCCN risk class 5 (ref: class 4)	0.6551 (0.1198-3.5822)	0.6256		
NCCN risk class 6 (ref: class 4)	3.4757 (1.1395–10.602)	**0.0286**		

* Final model selected by backward selection procedure: initial multivariate Cox regression model included as covariates age at diagnosis, T stage, Gleason score, diabetes, hypertension, previous abdominal surgery, ADT, and the categorized version of the initial PSA. Bold underlines statistically significant results.

**Table 4:** Acute and late toxicities and prevalence of late toxicities at last follow-up.

Grade	Acute uGE	Acute Rectal	Acute GU	Late GI	Late GU	Late Gl at Last Follow Up (%)	Late GU at Last Follow Up (%)
0	58%	71.9%	24.6%	67.4%	42.4%	198 (88.4)	141 (62.9)
1	34%	21%	46.4%	16.1%	29.5%	15 (6.7)	43 (19.2)
2	7.6%	7.1%	27.2%	8.0%	15.6%	4 (1.8)	22 (9.8)
3	0.4%	0	1.8%	8.5%	11.2%	7 (3.1)	15 (6.7)
4	0	0	0	0	1.3%		3 (1.3)

uGE = upper gastro-enteric, GU = genito-urinary, GI = gastro-intestinal (including uGE and rectal).

**Table 5:** Univariate and multivariate analysis of factors influencing toxicity.

**Late GU ≥ 3**	**Univariate (Cox Regression Model)**		Multivariate (Final * Selected Cox Regression Model)	
Variables	HR (95% CI)	*p*-value	HR (95% CI)	*p*-value
Age at diagnosis	0.9937 (0.9324–1.0592)	0.847	-	-
iPsa (log scale)	0.7653 (0.4524–1.2946)	0.3186	-	-
T stage T3/T4 (ref. T1 + T2)	0.6406 (0.1933–2.1228)	0.4663	-	-
Gleason score ≥ 8 (ref: ≤7)	0.6771 (0.3171–1.4459)	0.3137	-	-
ADT (yes vs. no)	0.9647 (0.3346–2.7817)	0.947	-	-
Abdominal surgery (yes vs. no)	1.1772 (0.5604–2.473)	0.6667	-	-
Hypertension (yes vs. no)	1.8579 (0.7897–4.3709)	0.1559	-	-
Diabetes (yes vs. no)	0.4664 (0.1107–1.9655)	0.2987	-	-
TURP (yes vs. no)	2.9464 (1.3795–6.2932)	**0.0053**	3.4919 (1.6179–7.5365)	**0.0014**
Acute GU toxicity ≥ G2 (ref: <2)	2.6187 (1.2481–5.4944)	**0.0109**	3.0755 (1.4508–6.5196)	**0.0034**
Acute GI (uGE + rectal) toxicity ≥ G2 (ref: <2)	1.785 (0.7237–4.4024)	0.2084	-	-
Neoadjuvant ADT (yes vs. no)	0.9262 (0.3519–2.4376)	0.8766		
Adjuvant ADT (yes vs. no)	0.4326 (0.1952–0.9585)	**0.039**		
NCCN risk class 5 (ref: class 4)	0.7506 (0.3109–1.8118)	0.5234		
NCCN risk class 6 (ref: class 4)	0.4476 (0.1786–1.1222)	0.0865		
Anticoagulant therapy (yes vs. no)	1.1223 (0.5254–2.3973)	0.7657		
**Late GI ≥ 3**	**Univariate (Cox Regression Model)**		Multivariate (Final* Selected Cox Regression model)	
Variables	HR (95% CI)	*p*-value	HR (95% CI)	*p*-value
Age at diagnosis	1.0612 (0.9729–1.1575)	0.1801	-	-
iPsa (log scale)	0.3642 (0.1739–0.7626)	**0.0074**	0.3677 (0.1624–0.8326)	**0.0164**
T stage T3/T4 (ref. T1 + T2)	1.0184 (0.2967–3.4962)	0.9769	-	-
Gleason score ≥ 8 (ref: ≤7)	0.7538 (0.3032–1.8741)	0.543	-	-
ADT (yes vs. no)	0.2632 (0.1036–0.669)	**0.005**	0.3104 (0.1208–0.7974)	**0.0151**
Abdominal surgery (yes vs. no)	1.7454 (0.7011–4.3453)	0.2313	-	-
Hypertension (yes vs. no)	3.3349 (0.9716–11.4471)	0.0556	3.6287 (1.0567–12.461)	**0.0406**
Diabetes (yes vs. no)	1.2158 (0.3542–4.1725)	0.7562	-	-
TURP (yes vs. no)	1.1442 (0.3797–3.4479)	0.8108	-	-
Acute GU toxicity ≥ G2 (ref: <2)	0.8837 (0.3183–2.4534)	0.8124	-	-
Acute GI (uGE + rectal) toxicity ≥ G2 (ref: <2)	1.2124 (0.3532–4.1612)	0.7596	-	-
Neoadjuvant ADT (yes vs. no)	0.2189 (0.0889–0.5392)	**0.001**		
Adjuvant ADT (yes vs. no)	0.2411 (0.0978–0.5944)	**0.002**		
NCCN risk class 5 (ref: class 4)	0.5904 (0.2018–1.7276)	0.3361		
NCCN risk class 6 (ref: class 4)	0.3292 (0.1033–1.0499)	0.0604		
Anticoagulant therapy (yes vs. no)	1.9585 (0.7955–4.8213)	0.1437		

The bold, to underline the statistically significant results.

## Data Availability

Anonymized individual participant data will be available following the publication of the article on a case-by-case basis to researchers who provide a methodologically sound proposal. Requests made to the corresponding author will be forwarded to be evaluated by the IRCCS San Raffaele Scientific Institute Ethics Committee.

## References

[1] Gray P.J., Zietman A.L. (2015). Dose-escalated radiotherapy for prostate cancer: Is the sky the limit?. JAMA Oncol..

[2] Dearnaley D.P., Jovic G., Syndikus I., Khoo V., Cowan R.A., Graham J.D., Aird E.G., Bottomley D., Huddart R.A., Jose C.C. (2014). Escalated-dose versus control-dose conformal radiotherapy for prostate cancer: Long-term results from the MRC RT01 randomised controlled trial. Lancet Oncol..

[3] Beckendorf V., Guerif S., Le Prisé E., Cosset J.-M., Bougnoux A., Chauvet B., Salem N., Chapet O., Bourdain S., Bachaud J.-M. (2011). 70 Gy Versus 80 Gy in Localized Prostate Cancer: 5-Year Results of GETUG 06 Randomized Trial. Int. J. Radiat Oncol. Biol. Phys..

[4] Zietman A.L., Bae K., Slater J.D., Shipley W.U., Efstathiou J.A., Coen J.J., Bush D.A., Lunt M., Spiegel D.Y., Skowronski R. (2010). Randomized Trial Comparing Conventional-Dose with High-Dose Conformal Radiation Therapy in Early-Stage Adenocarcinoma of the Prostate: Long-Term Results from Proton Radiation Oncology Group/American College of Radiology 95-09. J. Clin. Oncol..

[5] Kuban D.A., Tucker S.L., Dong L., Starkschall G., Huang E.H., Cheung M.R., Lee A.K., Pollack A. (2008). Long-Term Results of the M. D. Anderson Randomized Dose-Escalation Trial for Prostate Cancer. Int. J. Radiat. Oncol. Biol. Phys..

[6] Spratt D.E., Pei X., Yamada J., Kollmeier M.A., Cox B., Zelefsky M.J. (2013). Long-term Survival and Toxicity in Patients Treated with High-Dose Intensity Modulated Radiation Therapy for Localized Prostate Cancer. Int. J. Radiat. Oncol. Biol. Phys..

[7] Morgan S.C., Hoffman K., Loblaw D.A., Buyyounouski M.K., Patton C., Barocas D., Bentzen S., Chang M., Efstathiou J., Greany P. (2018). Hypofractionated Radiation Therapy for Localized Prostate Cancer: An ASTRO, ASCO, and AUA Evidence-Based Guideline. J. Clin. Oncol..

[8] Catton C.N., Lukka H., Gu C.-S., Martin J.M., Supiot S., Chung P.W.M., Bauman G.S., Bahary J.-P., Ahmed S., Cheung P. (2017). Randomized trial of a hypofractionated radiation regimen for the treatment of localized prostate cancer. J. Clin. Oncol..

[9] Arcangeli G., Saracino B., Arcangeli S., Gomellini S., Petrongari M.G., Sanguineti G., Strigari L. (2017). Moderate Hypofractionation in High-Risk, Organ-Confined Prostate Cancer: Final Results of a Phase III Randomized Trial. J. Clin. Oncol..

[10] Lee W.R., Dignam J.J., Amin M.B., Bruner D.W., Low D., Swanson G.P., Shah A.B., D’Souza D.P., Michalski J.M., Dayes I.S. (2016). Randomized Phase III Noninferiority Study Comparing Two Radiotherapy Fractionation Schedules in Patients with Low-Risk Prostate Cancer. J. Clin. Oncol..

[11] Dearnaley D., Syndikus I., Mossop H., Khoo V., Birtle A., Bloomfield D., Graham J., Kirkbride P., Logue J., Malik Z. (2016). Conventional versus hypofractionated high-dose intensity-modulated radiotherapy for prostate cancer: 5-year outcomes of the randomised, non-inferiority, phase 3 CHHiP trial. Lancet Oncol..

[12] Incrocci L., Wortel R.C., Alemayehu W.G., Aluwini S., Schimmel E., Krol S., van der Toorn P.-P., de Jager H., Heemsbergen W., Heijmen B. (2016). Hypofractionated versus conventionally fractionated radiotherapy for patients with localised prostate cancer (HYPRO): Final efficacy results from a randomised, multicentre, open-label, phase 3 trial. Lancet Oncol..

[13] Pollack A., Walker G., Horwitz E.M., Price R., Feigenberg S., Konski A.A., Stoyanova R., Movsas B., Greenberg R.E., Uzzo R.G. (2013). Randomized Trial of Hypofractionated External-Beam Radiotherapy for Prostate Cancer. J. Clin. Oncol..

[14] Yeoh E.E., Botten R.J., Butters J., -Di Matteo A.C., Holloway R.H., Fowler J. (2011). Hypofractionated versus conventionally fractionated radiotherapy for prostate carcinoma: Final results of phase III randomized trial. Int. J. Radiat. Oncol. Biol. Phys..

[15] Kuban D.A., Nogueras-Gonzalez G.M., Hamblin L., Lee A.K., Choi S., Frank S.J., Nguyen Q.N., Hoffman K.E., McGuire S.E., Munsell M.F. (2010). Preliminary Report of a Randomized Dose Escalation Trial for Prostate Cancer using Hypofractionation. Int. J. Radiat. Oncol. Biol. Phys..

[16] Lukka H., Hayter C., Julian J.A., Warde P., Morris W.J., Gospodarowicz M., Levine M., Sathya J., Choo R., Prichard H. (2005). Randomized Trial Comparing Two Fractionation Schedules for Patients with Localized Prostate Cancer. J. Clin. Oncol..

[17] Aluwini S., Pos F., Schimmel E., Krol S., van der Toorn P.P., de Jager H., Alemayehu W.G., Heemsbergen W., Heijmen B., Incrocci L. (2016). Hypofractionated versus conventionally fractionated radiotherapy for patients with prostate cancer (HYPRO): Late toxicity results from a randomised, non-inferiority, phase 3 trial. Lancet Oncol..

[18] Aluwini S., Pos F., Schimmel E., van Lin E., Krol S., van der Toorn P.P., de Jager H., Dirkx M., Alemayehu W.G., Heijmen B. (2015). Hypofractionated versus conventionally fractionated radiotherapy for patients with prostate cancer (HYPRO): Acute toxicity results from a randomised non-inferiority phase 3 trial. Lancet Oncol..

[19] Heidenreich A., Varga Z., Von Knobloch R. (2002). Extended pelvic lymphadenectomy in patients undergoing radical prostatectomy: High incidence of lymph node metastasis. J. Urol..

[20] Roach M., Moughan J., Lawton C.A.F., Dicker A.P., Zeitzer K.L., Gore E.M., Kwok Y., Seider M.J., Hsu I.C., Hartford A.C. (2018). Sequence of hormonal therapy and radiotherapy field size in unfavourable, localised prostate cancer (NRG/RTOG 9413): Long-term results of a randomised, phase 3 trial. Lancet Oncol..

[21] Pommier P., Chabaud S., Lagrange J.-L., Richaud P., Le Prise E., Wagner J.-P., Azria D., Beckendorf V., Suchaud J.-P., Bernier V. (2016). Is there a role for pelvic irradiation in localized prostate adenocarcinoma? Update of the long-term survival results of the GETUG-01 randomized study. Int. J. Radiat. Oncol. Biol. Phys..

[22] Di Muzio N.G., Fodor A., Noris Chiorda B., Broggi S., Mangili P., Valdagni R., Dell’Oca I., Pasetti M., Deantoni C.L., Chiara A. (2016). Moderate Hypofractionation with Simultaneous Integrated Boost in Prostate Cancer: Long-term Results of a Phase I-II Study. Clin. Oncol..

[23] Cozzarini C., Fiorino C., Di Muzio N., Alongi F., Broggi S., Cattaneo M., Montorsi F., Rigatti P., Calandrino R., Fazio F. (2007). Significant reduction of acute toxicity following pelvic irradiation with Helical Tomotherapy in patients with localized prostate cancer. Radiother. Oncol..

[24] Fodor A., Fiorino C., Picchio M., Di Muzio N. (2017). High-dose radiotherapy and pelvic lymph nodal irradiation for high-risk prostate cancer in the image-guided radiotherapy era. Re: Daniel E. Spratt, Herbert A. Vargas, Zachary S. Zumsteg.; et al. Patterns of lymph node failure after dose-escalated radiotherapy: Implications for extended pelvic lymph node coverage. Eur. Urol..

[25] Di Muzio N., Fiorino C., Cozzarini C., Alongi F., Broggi S., Mangili P., Guazzoni G., Valdagni R., Calandrino R., Fazio F. (2009). Phase I-II study of hypofractionated simultaneous integrated boost with tomotherapy for prostate cancer. Int. J. Radiat. Oncol. Biol. Phys..

[26] https://www.nccn.org/professionals/physician_gls/pdf/prostate.pdf.

[27] https://ctep.cancer.gov/protocolDevelopment/electronic_applications/docs/CTCAE_v5_Quick_Reference_5x7.pdf.

[28] Fiorino C., Alongi F., Broggi S., Cattaneo G.M., Cozzarini C., Di Muzio N., Maggiulli E., Mangili P., Perna L., Valdagni R. (2008). Physics aspects of prostate tomotherapy: Planning optimization and image-guidance issues. Acta Oncol..

[29] Fiorino C., Di Muzio N., Broggi S., Cozzarini C., Maggiulli E., Alongi F., Valdagni R., Fazio F., Calandrino R. (2008). Evidence of limited motion of the prostate by careful emptying the rectum as assessed by daily MVCT image-guidance with helical tomotherapy. Int. J. Radiat. Oncol. Biol. Phys..

[30] Fiorino C., Sanguineti G., Cozzarini C., Fellin G., Foppiano F., Menegotti L., Piazzolla A., Vavassori V., Valdagni R. (2003). Rectal dose-volume constraints in high-dose - radiotherapy for localized prostate cancer. Int. J. Radiat. Oncol. Biol. Phys..

[31] Roach M., Hanks G., Thames H., Schellhammer P., Shipley W.U., Sokol G.H., Sandler H. (2006). Defining biochemical failure following radiotherapy with or without hormonal therapy in men with clinically localized prostate cancer: Recommendations of the RTOG-ASTRO Phoenix Consensus Conference. Int. J. Radiat. Oncol. Biol. Phys..

[32] Arcangeli G., Arcangeli S., Pinzi V., Benassi M., Benassi M., Strigari L. (2018). Optimal scheduling of hypofractionated radiotherapy for localized prostate cancer: A systematic review and metanalysis of randomized clinical trials. Cancer Treat. Rev..

[33] Datta N.R., Stutz E., Rogers S., Bodis S. (2017). Conventional Versus Hypofractionated Radiation Therapy for Localized or Locally Advanced Prostate Cancer: A Systematic Review and Meta-analysis along with Therapeutic Implications. Int. J. Radiat. Oncol. Biol. Phys..

[34] de Vries K.C., Wortel R.C., Oomen-de Hoop E., Heemsbergen W.D., Pos F.J., Incrocci L. (2020). Hyprofractionated Versus Conventionally Fractionated Radiation Therapy for Patients with Intermediate- or High-Risk, Localized, Prostate Cancer: 7-Year Outcomes from the Randomized, Multicenter, Open-Label, Phase 3 HYPRO Trial. Int. J. Radiat. Oncol. Biol. Phys..

[35] Ekanger C., Helle S.I., Heinrich D., Johannessen D.C., Karlsdóttir Á., Nygård Y., Halvorsen O.J., Reisæter L., Kvåle R., Hysing L.B. (2019). Ten-Year Results from a Phase II Study on Image Guided, Intensity Modulated Radiation Therapy With Simultaneous Integrated Boost in High-Risk Prostate Cancer. Adv. Radiat. Oncol..

[36] Abu-Gheida I., Reddy C.A., Kotecha R., Weller M.A., Shah C., Kupelian P.A., Mian O., Ciezki J.P., Stephans K.L., Tendulkar R.D. (2019). Ten-Year Outcomes of Moderately Hypofractionated (70 Gy in 28 fractions) Intensity Modulated Radiation Therapy for Localized Prostate Cancer. Int. J. Radiat. Oncol. Biol. Phys..

[37] Murthy V., Maitre P., Kannan S., Panigrahi G., Krishnatry R., Bakshi G., Prakash G., Pal M., Menon S., Phurailatpam R. (2021). Prostate-Only Versus Whole-Pelvic Radiation Therapy in High-Risk and Very High-Risk Prostate Cancer (POP-RT): Outcomes From Phase III Randomized Controlled Trial. J. Clin. Oncol..

[38] Faria S., Cury F., Duclos M., Souhami L. (2018). Hypofractionated Intensity Modulated Radiation Therapy to Prostate and Pelvic Nodes Plus Androgen Suppression in High-Risk Prostate Cancer. Int. J. Radiat. Oncol. Biol. Phys..

[39] Amini A., Jones B.L., Yeh N., Rusthoven C.G., Armstrong H., Kavanagh B.D. (2015). Survival Outcomes of Whole-Pelvic Versus Prostate-Only Radiation Therapy for High-Risk Prostate Cancer Patients with Use of the National Cancer Data Base. Int. J. Radiat. Oncol. Biol. Phys..

[40] Lawton C.A., DeSilvio M., Roach M., Uhl V., Kirsch R., Seider M., Rotman M., Jones C., Asbell S., Valicenti R. (2007). An update of the phase III trial comparing whole pelvic to prostate only radiotherapy and neoadjuvant to adjuvant total androgen suppression: Updated analysis of RTOG 94–13, with emphasis on unexpected hormone/radiation interactions. Int. J. Radiat. Oncol. Biol. Phys..

[41] Horwitz E.M., Bae K., Hanks G.E., Porter A., Grignon D.J., Brereton H.D., Venkatesan V., Lawton C.A., Rosenthal S.A., Sandler H.M. (2008). Ten-Year Follow-Up of Radiation Therapy Oncology Group Protocol 92-02: A Phase III Trial of the Duration of Elective Androgen Deprivation in Locally Advanced Prostate Cancer. J. Clin. Oncol..

[42] Roach M., Bae K., Speight J., Wolkow H.B., Rubin P., Lee R.J., Lawton C., Valicenti R., Grignon D., Pilepich M.V. (2008). Short-term neoadjuvant androgen deprivation therapy and external-beam radiotherapy for locally advanced prostate cancer: Long-term results of RTOG 8610. J. Clin. Oncol..

[43] Mercader M., Bodner B.K., Moser M.T., Kwon P.S., Park E.S., Manecke R.G., Ellis T.M., Wojcik E.M., Yang D., Flanigan R.C. (2001). T cell infiltration of the prostate induced by androgen withdrawal in patients with prostate cancer. Proc. Natl. Acad. Sci. USA.

[44] Jiang T., Markovic D., Patel J., Juarez J.E., Ma T.M., Shabsovich D., Nickols N.G., Reiter R.E., Elashoff D., Rettig M.B. (2021). Radiation therapy dose and androgen deprivation therapy in localized prostate cancer: A meta-regression of 5-year outcomes in phase III randomized controlled trials. Prostate Cancer Prostatic Dis..

[45] Kishan A.U., Cook R.R., Ciezki J.P., Ross A.E., Pomerantz M., Nguyen P.L., Shaikh T., Tran P.T., Sandler K.A., Stock R.G. (2018). Radical prostatectomy, external beam radiotherapy, or external beam radiotherapy with brachytherapy boost and disease progression and mortality in patients with Gleason Score 9–10 prostate cancer. JAMA.

[46] Martinez A.A., Gonzalez J., Ye H., Ghilezan M., Shetty S., Kernen K., Gustafson G., Krauss D., Vicini F., Kestin L. (2011). Dose Escalation Improves Cancer-Related Events at 10 Years for Intermediate- and High-Risk Prostate Cancer Patients Treated with Hypofractionated High-Dose-Rate Boost and External Beam Radiotherapy. Int. J. Radiat. Oncol. Biol. Phys..

[47] Wedde T.B., Smastuen M.C., Brabrand S., Fossa S.D., Kaasa S., Tafjord G., Russnes K.M., Hellebust T.P., Lilleby W. (2019). Ten-year survival after high-dose-rate brachytherapy combined with external beam radiation therapy in high-risk prostate cancer: A comparison with the Norwegian SPCG-7 cohort. Radiother. Oncol..

[48] Saracino B., Petrongari M.G., Marzi S., Bruzzaniti V., Gomellini S., Arcangeli S., Arcangeli G., Pinnarò P., Giordano C., Ferraro A.M. (2014). Intensity-modulated pelvic radiation therapy and simultaneous integrated boost to the prostate area in patients with high-risk prostate cancer: A preliminary report of disease control. Cancer Med..

[49] Zapatero A., Guerrero A., Maldonado X., Alvarez A., Gonzalez San Segundo C., Cabeza Rodriguez M.A., Macias V., Olive A.P., Casas F., Boladeras A. (2015). High-dose radiotherapy with short-term or long-term androgen deprivation in localized prostate cancer (DART01/05 GICOR): A randomized, controlled, phase 3 trial. Lancet Oncol..

[50] Spratt D.E., Vargas H.A., Zumsteg Z.S., Golia Pernicka J.S., Osborne J.R., Pei X., Zelefsky M.J. (2017). Patterns of lymph node failure after dose-escalated radiotherapy: Implications for extended pelvic lymph node coverage. Eur. Urol..

[51] Roach M., DeSilvio M., Valicenti R., Grignon D., Asbell S.O., Lawton C., Thomas C.R., Shipley W.U. (2006). Whole-pelvis, “mini-pelvis,” or prostate-only external beam radiotherapy after neoadjuvant and concurrent hormonal therapy in patients treated in the Radiation Therapy Oncology Group 9413 trial. Int. J. Radiat. Oncol..

[52] Hope T.A., Eiber M., Armstrong W.R., Juarez R., Murthy V., Lawhn-Heath C., Behr S.C., Zhang L., Barbato F., Ceci F. (2021). Diagnostic Accuracy of 68Ga-PSMA-11 PET for Pelvic Nodal Metastasis Detection Prior to Radical Prostatectomy and Pelvic Lymph Node Dissection. A multicenter prospective phase 3 imaging trial. JAMA Oncol..

[53] Pienta K.J., Gorin M.A., Rowe S.P., Caroll P.R., Pouliot F., Probst S., Saperstein L., Preston M.A., Alva A.S., Patnaik A. (2021). A phase 2/3 prospective multicenter study of the diagnostic accuracy of Prostate Specific Membrane Antigen PET/CT with 18F-DCFPyL in prostate cancer patients (OSPREY). J. Urol..

[54] Widmark A., Gunnlaugsson A., Beckmann L., Thellenberg-Karlsson C., Hoyer M., Lagerlund M., Kindblom J., Ginman C., Johansson B., Bjornlinger K. (2019). Ultrahypofractionated versus conventionally fractionated radiotherapy for prostate cancer: 5-year outcomes of the HYPO-RT-PC randomised, non-inferiority, phase 3 trial. Lancet.

[55] Kishan A.U., Dang A., Katz A.J., Mantz C.A., Collins S.P., Aghdam N., Chu F.-I., Kaplan I.D., Appelbaum L., Fuller D.B. (2019). Long-term Outcomes of Stereotactic Body Radiotherapy for Low-Risk and Intermediate-Risk Prostate Cancer. JAMA Netw. Open.

[56] Jackson W.C., Silva J., Hartman H.E., Dess R.T., Kishan A.U., Beeler W.H., Gharzai L.A., Jaworski E.M., Mehra R., Hearn J.W.D. (2019). Stereotactic Body Radiotherapy for Localized Prostate Can-cer: A Systematic Review and Meta-Analysis of Over 6000 Patients Treated on Prospective Studies. Int. J. Radiat. Oncol. Biol. Phys..

[57] Krebs M., Solimando A.G., Kalogirou C., Marquardt A., Frank T., Sokolakis I., Hatzichristodoulou G., Kneitz S., Bargou R., Kübler H. (2020). miR-221-3p Regulates VEGFR2 Expression in High-Risk Prostate Cancer and Represents an Escape Mechanism from Sunitinib In Vitro. J. Clin. Med..

[58] Cucchiara V., Cooperberg M.R., Dall’Era M., Lin D.W., Montorsi F., Schalken J.A., Evans C.P. (2018). Genomic Markers in Prostate Cancer Decision Making. Eur. Urol..

